# Treatment of Diarrhœa by Chloric Ether

**Published:** 1855-01

**Authors:** George B. Mead


TREATMENT OF DIARRHŒA BY CHLORIC ETHER. BY GEORGE B. MEAD, Esq.—We often find that many cases of diarrhœa resist all ordinary treatment, opium amongst the iest. In 1846, '7, '8, an epidemic prevailed at Bradford, Yorkshire, which was attended by a diarrhœa of an intractable nature.

This diarrhœa was combined in many cases with vomiting and spasmodic pain of a very distressing character. We had very little difficulty in treating those cases in which pain was absent, but we found a certain proportion in which the diarrhœa obstinately continued despite the use of a multitude of remedies; and the pains, though temporarily relieved by the use of opium, returned directly the narcotism passed off; the opium suspended, but did not remove, the spasms. At length we adopted the following formula:—

| | | |
|---|---|---|
| ℞. | Ætheris chlorici, | ʒij, |
| | Speciei pro conf. arom., | ʒss, |
| | Misturæ cretæ compositæ ad | ℥vj, |
| | M. Fiat mistura. | |

The fourth part was directed to be taken directly by an adult, and repeated every half hour, or at still longer intervals, according to the severity of the attack, and its effect upon the patient. Occasionally, opium, either in the solid form or the tincture, added to the mixture, was given; but this was seldom necessary, and I think every case would have recovered without its use. The effect of the ether in every case was marvellous. The spasms and pains were relieved, as if by a charm; the diarrhœa ceased; warmth returned to the extremities; the pulse, before perhaps flagging, increased in force and volume; one bottle never failed to relieve, or two to cure even the worst cases in which all other plans had failed. The relapses were infrequent, and were generally checked at once by a single dose. After the introduction of the chloric ether, we had no further trouble with diarrhœa. The medical man in whose practice this occurred is since dead; otherwise I am sure he would have felt great pleasure in confirming this statement. Often has he expressed to me his high opinion of the efficacy of the ether in cases of diarrhœa combined with spasmodic pain. After this, at the time the cholera last visited this country, I was residing with Dr Morris, of Spalding. It reached Boston. Great alarm existed in Spalding, and the public were fully aware of the importance of checking the premonitory diarrhœa which began to prevail, and was exceedingly troublesome in many cases to check. Recollecting the ether, I ventured to suggest its use. My friend acquiesced. It was tried, and in not one case failed. As at Bradford, hundreds of cases in which alarming cramps existed were cured like magic; none ran on into Asiatic cholera, though many appeared to be on the verge of it, or, at any rate, were equal to those of English cholera of the severest character. So prevalent was the diarrhœa, that the surgery was thronged from morning to night by applicants for medicine; the policemen in the streets, sailors in the vessels on the river, travellers upon the rail, were equally affected; the cause was evidently ubiquitous, whatever it might be.

At last, one morning early, I was summoned in haste to a patient, an old woman, residing in one of the most unhealthy localities in Spalding. I found her presenting unmistakeable signs of laboring under Asiatic cholera in an advanced stage; she was blue, almost pulseless, and the cramps were of a most violent character. I had seen English cholera in its worst and most fatal forms, but never aught like this. I have a vivid recollection of the scene, and remember well I stood as it were aghast at the sight of her agonies. Having directed her attendants how to act, I instantly returned home, and sent a mixture, increasing the dose of either, but with very little hope of success. I ordered it to be given at very short intervals, and, in the course of an hour or two, saw her again, in company with my friend. We were delighted and surprised to find her greatly improved. We continued the remedies, and by the evening she was out of danger. Symptoms of relapse appeared several times during the next day or two, but were at once checked by the use of the remedy. The patient, though advanced in years and greatly debilitated, having led a somewhat irregular life, completely recovered, and died about a year afterwards, of a totally different complaint. The remedy obtained quite a notoriety in that locality; to such an extent was it used, that we were told by an extensive wholesale druggist he had sold more to us than to all the rest of his connection put together.

A gentleman travelling by the Great Northern Railway was attacked by severe diarrhœa. He got out at Spalding, and consulted Dr. Morris, who prescribed this remedy; it at once relieved him. In conversation, he mentioned to him how valuable he had found it in the treatment of diarrhœa. On his return home, the gentleman told his usual medical man of the circumstance; and he, in consequence, was induced to try it, with great success.

In 1850 and 1851, I was again residing in Yorkshire, and there saw much diarrhœa. I used the ether extensively, and never found any case in which it was properly administered where it failed; indeed, I recollect some equally striking cases with those I have enumerated, in which it might indeed be said to have acted like a charm. I could refer to many of my medical friends who have used it upon my recommendation, and whose experience after numerous trials, has strongly corroborated my own. Though I have used it in at least fifteen hundred cases myself, and indeed I think not improbably three thousand cases, I have never yet found it fail.—*Association Medical Journal.*

